# Threshold Adaptation for Improved Wrapper-Based Evolutionary Feature Selection

**DOI:** 10.3390/biomimetics10100670

**Published:** 2025-10-05

**Authors:** Uroš Mlakar, Iztok Fister, Iztok Fister

**Affiliations:** Faculty of Electrical Engineering and Computer Science, University of Maribor, Koroška Cesta 46, 2000 Maribor, Slovenia

**Keywords:** feature selection, evolutionary algorithm, feature threshold, evolutionary feature selection

## Abstract

Feature selection is essential for enhancing classification accuracy, reducing overfitting, and improving interpretability in high-dimensional datasets. Evolutionary Feature Selection (EFS) methods employ a threshold parameter θ to decide feature inclusion, yet the widely used static setting θ=0.5 may not yield optimal results. This paper presents the first large-scale, systematic evaluation of threshold adaptation mechanisms in wrapper-based EFS across a diverse number of benchmark datasets. We examine deterministic, adaptive, and self-adaptive threshold parameter control under a unified framework, which can be used in an arbitrary bio-inspired algorithm. Extensive experiments and statistical analyses of classification accuracy, feature subset size, and convergence properties demonstrate that adaptive mechanisms outperform the static threshold parameter control significantly. In particular, they not only provide superior tradeoffs between accuracy and subset size but also surpass the state-of-the-art feature selection methods on multiple benchmarks. Our findings highlight the critical role of threshold adaptation in EFS and establish practical guidelines for its effective application.

## 1. Introduction

Feature selection is a critical step in data preprocessing, especially in high-dimensional datasets often encountered in fields such as bioinformatics [1] and data mining [2]. The goal of feature selection is to identify a subset of relevant features that contribute the most to the predictive power of a model, thereby enhancing performance, reducing overfitting, and improving interpretability. Among the various feature selection methods, bio-inspired algorithms have gained significant attention due to their ability to search large and complex spaces efficiently. Additionally, hybrid approaches have been developed by combining evolutionary algorithms with other simpler optimization techniques, such as Simulated Annealing [3] or traditional filter methods [4], to balance exploration and exploitation more effectively. These hybrid methods often result in faster convergence and improved accuracy, as they capitalize on the strengths of each individual algorithm.

Evolutionary Feature Selection (EFS) methods, which apply bio-inspired algorithms, like evolutionary algorithms (EAs) [5] and Swarm Intelligence (SI)-based algorithms [6], to the task of selecting the optimal subset of features have shown great promise in handling high-dimensional data. These methods rely on the models of biology (like natural selection, the behavior of bird swarms, flocks of fishes, etc.) to evolve feature subsets iteratively, balancing between exploration and exploitation to avoid the local optima. The balancing depends crucially on a parameter threshold that determines the inclusion/exclusion of the particular feature in the solution subset. Indeed, this parameter influences the tradeoff between model complexity and generalization capability. The higher threshold might result in a smaller subset, potentially leading to underfitting if relevant features are discarded. Conversely, the lower threshold might retain too many features, increasing the risk of overfitting and computational cost. Therefore, finding the optimal threshold is essential for maximizing the effectiveness of EFS methods.

Recent works have explored various aspects of EFS, such as the development of new crossover and mutation strategies, hybrid approaches combining EFS with other optimization techniques, and the application of EFS in different domains. Wang et al. [1] introduced a PSO-based feature selection algorithm with a dynamic adjustment mechanism for the inertia weight, enhancing convergence speed and solution quality. Many studies highlight the potential of EFS methods in feature selection, but often overlook the impact of the threshold parameter, focusing primarily on algorithmic innovations. Moreover, some recent studies have recognized the need to optimize feature selection thresholds but have approached it from a heuristic or domain-specific angle. Deng et al. [7] proposed a novel approach for high-dimensional feature selection, named the Feature-Thresholds-Guided Genetic Algorithm (FTGGA). Traditional genetic algorithms suffer from unguided crossover and mutation operations, leading to slow convergence and suboptimal features. To address these challenges, FTGGA introduces a multi-objective feature scoring mechanism that updates feature thresholds during the evolutionary process, allowing for a more targeted crossover and mutation. However, the algorithm integrates the ReliefF [8] technique to filter out most of the redundant features initially, followed by a genetic algorithm guided by continuously updated feature thresholds. Li et al. [9] proposed an Improved Sticky Binary Particle Swarm Optimization (ISBPSO) algorithm for feature selection in high-dimensional classification tasks. The method enhances the standard SBPSO by integrating three key mechanisms: a feature-weighted initialization using mutual information, a dynamic bit masking strategy that reduces the search space progressively by freezing unpromising features, and a genetic refinement process applied to the particles’ personal bests to prevent premature convergence. A notable distinction of ISBPSO is the use of a feature selection threshold of 0.6 instead of the typical 0.5, in order to retain only strongly activated features. Fister et al. [10] introduced a novel Self-Adaptive Differential Evolution Algorithm for feature selection, enhanced by a threshold mechanism. Their approach improved feature selection by updating feature presence thresholds dynamically by means of complex adaptation during the evolutionary process.

In contrast to these method-specific designs, our study tests various threshold parameter control mechanisms systematically under a unified, optimizer-agnostic framework. According to Eiben and Smith [5], the parameter control techniques in evolutionary computation can be classified into one of the following three categories: (1) deterministic, (2) adaptive, and (3) self-adaptive. The algorithm’s parameters are altered according to some deterministic rule by the deterministic parameter control. The adaptive parameter control means that there is some feedback from the search process, which determines the direction or magnitude of the change by the control parameters. In the last parameter control mechanism, the parameters are included into the representation of individuals, and, together with the problem variables, they suffer the effects of the variation operators. In this sense, we benchmark deterministic schedules, population-level feedback mechanisms, and self-adaptive per-individual threshold adaptations across multiple bio-inspired optimizers and datasets, using a common interface and evaluation protocol. This isolates the mechanism effect of threshold adaptation from confounding factors (e.g., prefiltering or operator choice) and yields generalizable guidance on when and how adaptive thresholding improves the quality of the selected feature subsets. In order to present a picture as comprehensively as possible, the random search deterministic algorithm with no parameter control is included into the comparative study.

To the best of our knowledge, this study represents the first large-scale systematic investigation of several mechanisms for feature selection threshold adaptation across a wide range of bio-inspired algorithms and datasets. This allows us to uncover generalizable insights about threshold behavior that are independent of the underlying evolutionary operator. The obtained results suggest that using a higher static threshold already achieves significant improvements in classification accuracy and subset compactness, highlighting the importance of tuning the feature threshold. To summarize, the proposed paper introduces the following key novelties:

- Proposing a threshold adaptation mechanism, which can be used in an arbitrary bio-inspired algorithm for EFS;
- Comparing different feature threshold adaptation mechanisms to the baseline method (θ = 0.5);
- Investigating the balance of classification accuracy and feature subset size in the fitness function by using five different threshold mechanisms in bio-inspired algorithms;
- A large-scale study of five feature threshold adaptation mechanisms in bio-inspired algorithms and their influence on the quality of the selected feature subset;
- A large-scale study of five feature threshold adaptation mechanisms in bio-inspired algorithms and their influence on the size of the selected feature subset;
- Investigating the convergence properties of the bio-inspired algorithm in regard to using different feature threshold adaptation mechanisms;
- Comparing the best adaptation mechanism (according to the obtained results) to the state of the art.

The rest of the paper is organized as follows: In Section 2, the foundation of EFS is explained in detail. Section 3 illustrates the design and implementation of the proposed method. The experimental work and the analysis of the obtained results are the subjects of Section 4. Finally, the paper is concluded with Section 5, where we explain the potential directions of future work.

## 2. Materials and Methods

This section introduces the foundational knowledge necessary for potential readers to grasp to understand the concepts that follow. Firstly, the feature selection problem is defined, which is presented as an optimization problem. Then, the idea of wrapper-based feature selection is introduced. Finally, the application of a wrapper-based feature selection is defined using an arbitrary evolutionary algorithm.

### 2.1. Feature Selection

Feature selection is a preprocessing mechanism, which involves identifying the most relevant subset of features from a given dataset, thereby reducing the dimension of the problem and improving the learning algorithm efficiency and performance. Mathematically, the feature selection problem can be formulated as follows: Let $X=\{F_{1},F_{2},\ldots ,F_{n}\}$ represent the set of all *n* features in a dataset, and let *Y* denote the corresponding target class variable. The goal of feature selection is to find a subset of features S⊆X, such that a model trained on *S* achieves optimal performance in terms of an evaluation metric f(S). Formally, the feature selection is expressed as follows:

(1) argminf(S):{S⊆X},

where f(S) represents the performance of the trained model on the selected feature subset *S* and depends on the selected feature selection method.

### 2.2. Evolutionary Feature Selection

EFS is a bio-inspired optimization approach in order to identify the most relevant subset of features. Unlike traditional methods, which often rely on deterministic algorithms, bio-inspired computation comprises a class of stochastic nature-inspired population-based search algorithms suitable for solving the hardest optimization problems. These algorithms evolve a population of candidate feature subsets iteratively, by selecting, combining, and mutating them to explore the search space effectively. This process not only enhances model performance by reducing overfitting, but also improves interpretability and computational efficiency by eliminating irrelevant or redundant features. EFS is particularly useful in high-dimensional datasets where the feature space is vast, making an exhaustive search impractical.

During the optimization, a bio-inspired algorithm maintains a population of solutions $x=\{x_{1},x_{2},\ldots ,x_{i}\}$, for i=1,…,Np, where Np denotes the population size. Each solution $x_{i}=\left\{x_{i,1},x_{i,2},\ldots ,x_{i,j}\right\}$ is a vector of *j* values, where *j* corresponds to the number of all features in the dataset. Each element of the vector $x_{i,j}$ represents a feature from the dataset. All of the reviewed bio-inspired algorithms for feature selection use a threshold mechanism internally for selecting the relevant features in the search space of the algorithm. This can be expressed mathematically as follows:

(2) $$S=\{\forall x_{j}:x_{j}\geq \theta \},$$

where the variable *S* denotes the feature subset, which will be used for training the selected machine learning algorithm, and the parameter θ is a threshold determining if the specific feature will be included in the feature subset or not. Let us mention that the value of the threshold θ is typically set to 0.5 in most of the reviewed literature.

### 2.3. Wrapper-Based Feature Selection

Wrapper-based methods evaluate the usefulness of feature subsets by using the predictive model as a black box to assess their performance directly. By iterative searching and selecting subsets of features that optimize a given performance metric, wrapper-based approaches can identify the most relevant features for the model effectively. This method is computationally intensive but often yields superior results compared to filter-based methods, as it considers feature dependencies and interactions within the context of the specific predictive algorithm. The wrapper-based feature selection process in our study is implemented using a bio-inspired algorithm, which evolves a population of feature subsets iteratively towards optimal solutions inspired by models which have arisen in biology.

The fitness function for evaluating a solution in a wrapper-based method is defined as follows:

(3) Acc=Classify(C,S),

where the variable *C* designates the selected classifier and the variable *S* is the feature subset. The variable Acc∈[0,1] denotes the classification accuracy of the observed feature subset *S*.

Since the feature subset size also plays an important role, the following fitness function was adapted in this study:

(4) $$f\left(S\right)=\beta \left(1-Acc\right)+\left(1-\beta \right)\frac{L(S)}{L(X)},$$

where L(·) is the length function, which counts the number of features in a subset *S*, and β is the weighing factor for balancing the importance between the classification accuracy and the number of selected features.

## 3. The Proposed Evolutionary Feature Selection and Threshold Adaptation Mechanisms

**Figure 1:**
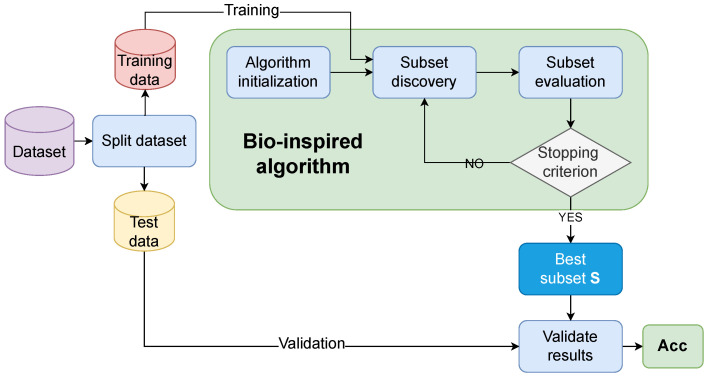
General process of EFS.

This section describes the complete EFS framework used throughout our experiments. The framework builds upon a generic bio-inspired optimization algorithm using a threshold-based genotype–phenotype mapping to control feature subset generation. It is implemented as a wrapper-based approach and is compatible with an arbitrary population-based bio-inspired algorithm (Figure 1).

As is evident from Figure 1, a general feature selection process is divided into the following four key steps:

- Dataset splitting;
- Subset discovery;
- Subset evaluation;
- Validation of results.

In the first step (i.e., dataset splitting), a dataset is divided into training and validation sets with respect to some predefined ratio. Thus, the former set is used for the training phase, while the latter is used for the validation phase of the EFS. The second step (i.e., subset discovery) involves generating candidate subsets of features from the full feature space. In the context of evolutionary algorithms, this corresponds to evolving a population of individuals where each individual encodes a potential feature subset. The third step (i.e., the subset evaluation) is achieved by applying a fitness function to assess the quality of each feature subset. The last step (i.e., validation of results) evaluates the selected subset on a separate testing set to assess its generalization performance. This step is crucial to avoid overfitting and to ensure the robustness of the selected features across different data splits. The result of the process is the best subset *S* according to the fitness value, as proposed by the definite bio-inspired algorithm and its accuracy Acc.

Let us mention that the second and third steps are entrusted to a particular bio-inspired algorithm by the framework. Although the concept of bio-inspired algorithms captures two classes of nature-inspired algorithms (EAs and SI-based), they share common characteristics that enable us to deal with them similarly. Moreover, some efforts were made by Fister et al. in defining the universal framework of these stochastic nature-inspired population-based algorithms [11]. As a result, the generic bio-inspired algorithm can be defined as illustrated in the pseudo-code of Algorithm 1.
**Algorithm 1** The pseudo-code of a generic bio-inspired algorithm.1:INITIALIZE_population_randomly2:EVALUATE_each_individual3:**while** Termination_condition_not_met **do**4:      MODIFY_each_individual5:      EVALUATE_each_trial6:      SELECT_individuals_for_the_next_generation7:      FIND_global_best_individual8:**end while**

Indeed, these algorithms follow a common evolutionary paradigm and differ only in their specific update/modification mechanisms (i.e., function ‘MODIFY_each_individual’). In summary, the function in EAs consists of the following three functions [5]:

- SELECT_parents;
- RECOMBINE_pairs_of_parents;
- MUTATE_the_resulting_offspring.

However, in SI-based algorithms, this function represents the implementation of some biological model that serves as an inspiration for the search process design captured in the ‘MODIFY_each_individual’ function. Therefore, our main effort in the design was to adapt the specific bio-inspired algorithm to be capable of solving the feature selection problem as an optimization. Indeed, the adaptation demands two modifications of the bio-inspired algorithm, namely the following:

- genotype–phenotype mapping;
- fitness function evaluation.

**Figure 2:**
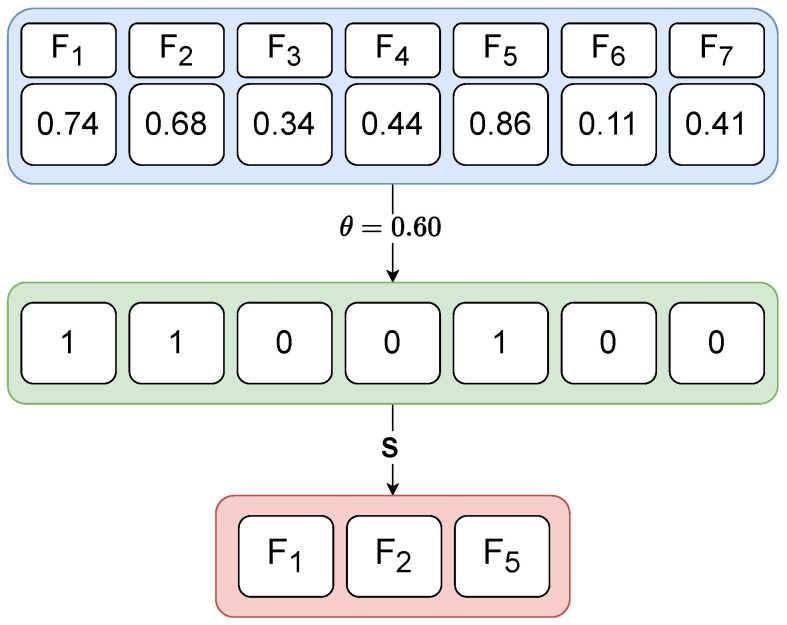
Example of a genotype–phenotype mapping of an individual using the feature threshold. This example depicts the feature selection process on a dataset with 7 features, and a threshold θ=0.60. The selected feature subset *S* according to the threshold is $\left\{F_{1},F_{2},F_{5}\right\}$.

The genotype–phenotype mapping decodes the representation of an encoded solution in the search space to the solution in the problem space. The solution of the EFS in the genotype search space is represented as a real-valued vector of length equal to the number of features, while, in the phenotype space, this is decoded as a binary feature mask derived by applying a threshold to the genotype vector. Specifically, features with values above the threshold θ are included in the selected subset, while others are excluded (see Equation (2) and Figure 2).

In our study, a wrapper-based approach is used, where the fitness function considers the classification accuracy and the number of selected features jointly (see Equation (4)).

### Threshold Parameter Control

To study the control of the threshold parameter systematically, we grouped the different parameter control mechanisms into three classes that can be applied to an arbitrary bio-inspired algorithm:

1. Deterministic schedules, varying the threshold over time according to a preset curriculum (e.g., linear ramps, cosine cycles), shaping feature selection pressure without using any feedback from the population;
2. Population-level feedback mechanisms updating a single global threshold by regulating the measurable metrics, such as improvement/success rate or diversity, thereby tightening or relaxing selection as the search progresses;
3. Self-adaptive per-individual thresholds treating the threshold as a gene, which is co-evolved with the features, allowing different individuals to investigate the search space on their own.

All the used mechanisms exposed the same interface, which consumes population summaries and then outputs the global threshold parameter $\theta_{t}$ or the local, i.e., per-individual-based threshold parameter $\theta_{i,t}$, so they can be compared fairly and used across all bio-inspired methods under the common objective.

Two deterministic threshold parameter control mechanisms were used, namely, Linear Ramp (LR) and Cosine Ramp (CR). The LR increases the threshold linearly over generations as follows:

(5) $$\theta_{t}\;=\;\theta_{\min}\;+\;\frac{t}{T}\left(\theta_{\max}-\theta_{\min}\right),\;t=0,\ldots ,T,$$

while the CR increases the threshold by following a half-cosine curve, thus ensuring a slow increase at the beginning and end, with a faster transition in the middle; in other words,

(6) $$\theta_{t}\;=\;\theta_{\min}\;+\;\frac{\theta_{\max}-\theta_{\min}}{2}\left(1-\cos\frac{\pi \;t}{T}\right),$$

where *t* denotes the current generation, *T* the maximum number of generations, and $\theta_{\min}$ and $\theta_{\max}$ are the minimum and the maximum values of the threshold parameter.

We implemented two adaptive threshold parameter control mechanisms: The first mechanism, called Proportional Control (PC), regulates the threshold parameter toward a target feature subset size. It uses a target selection rate $\rho^{★}$, which represents the desired fraction of selected features. At each generation *t*, we compute the realized selection rate $\rho_{t}$ from the population and form the error rate $e_{t}=\rho_{t}-\rho^{★}$. The threshold is then updated with a learning rate η>0. The larger positive error rate leads to the larger increase in the threshold, which yields the smaller feature subset in the next generation, while the negative error rate produces the opposite effect. The projection operator $\Pi_{\left[\theta_{\min},\theta_{\max}\right]}\left(\cdot \right)$ keeps the threshold parameter within the desired interval $\left[\theta_{\min},\theta_{\max}\right]$, i.e.,

(7) $$\theta_{t+1}=\Pi_{\left[\theta_{\min},\theta_{\max}\right]}\;\left(\theta_{t}+\eta \;\left(\rho_{t}-\rho^{★}\right)\right).$$

The second mechanism, called Success Rate Adaptation (SRA), adjusts the threshold parameter $\theta_{t+1}$ using the fraction of individuals improving their fitness in the current generation. For instance, let $SR_{t}\in \left[0,1\right]$ denote the success rate and $SR^{★}=0.15$ be the target success rate, which is determined empirically. When the observed success rate falls below the target one, the threshold is increased by 15%, to promote smaller subsets and stronger exploitation. When the observed success rate exceeds the target, the threshold is decreased by 15%, to promote larger subsets and additional exploration; in other words,

(8) $$\begin{array}{cc} SR_{t} & =\frac{1}{N}\sum_{i=1}^{N}\left[f{\left(x_{i}\right)}_{t}>f{\left(x_{i}\right)}_{t-1}\right], \end{array}$$

(9) $$\begin{array}{cc} \theta_{t+1} & =\Pi_{\left[\theta_{\min},\theta_{\max}\right]}\left\{\begin{array}{cc} \theta_{t}\cdot c_{↓}, & {\mathrm{SR}}_{t}>SR^{★},\\ \theta_{t}\cdot c_{↑}, & {\mathrm{SR}}_{t}\leq SR^{★}, \end{array}\right.\; \end{array}$$

where $c_{↓}=0.85$ and $c_{↑}=1.15$ are constants which control the value of the threshold $\theta_{t+1}$ in the new generation.

The final parameter control mechanism, called Self-Adaptation (SA), assigns each individual its own threshold parameter $\theta_{i}\in \left[\theta_{\min},\theta_{\max}\right]$. This parameter is encoded in the genome and updated by the variation operators of the evolutionary search process together with the problem variables. Each individual therefore operates with its own selection rate and can adjust it over time. Different solutions explore different areas of the search space at the same time, which increases population diversity and allows each individual to progress at its own pace without relying on a single global controller. Thus, the $\theta_{i}$ threshold control parameter becomes a part of solution; in other words,

(10) $$x_{i}=\left(x_{i,1},\ldots ,x_{i,N},\;\theta_{i}\right),$$

where $x_{i,j}$ for j=1,…,N denote the problem variables, and *N* is the number of elements. Thus, each individual produces the feature subset $S_{i}$ using their own threshold, as follows:

(11) $$S_{i}=\left\{\forall x_{i,j}:x_{i,j}\geq \theta_{i}\right\},\;\mathrm{for}\;j=1,\ldots ,N.$$

## 4. Experiments and Results

This section describes the results of the experimental work which was conducted in this study. The main goal of the experimental work was to check whether the threshold value of the feature selection process in the search space of the algorithm has any implications on the quality of the feature selection process. In line with this, the following experiments were conducted:

- Determining the best baseline bio-inspired algorithm;
- Investigating the impact of different threshold parameter control mechanisms on the classification accuracy;
- Investigating the impact of different threshold parameter control mechanisms on the feature subset size;
- Analyzing the algorithm’s convergence;
- Comparing the best parameter control mechanism with state-of-the-art algorithms.

Although various evolutionary algorithms have been proposed and refined to address the challenges of feature selection, we selected a broad set of both classical and state-of-the-art bio-inspired algorithms for our experimental work. Specifically, we considered Differential Evolution (DE) [12], Particle Swarm Optimization (PSO) [13], Self-Adaptive Differential Evolution (jDE) [14], Linear Population Reduction Success History Adaptive Differential Evolution (LSHADE) [15], genetic algorithm (GA) [7], and Artificial Bee Colony algorithm (ABC) [16]. These algorithms were chosen because they represent the most widely used methods in Evolutionary Feature Selection, as reported in recent surveys [17,18,19], and have also been shown to perform well in related studies (e.g., LSHADE in the CEC competition). To provide a baseline and to cover fixed parameter strategies, we further included random search (RS) [20]. In line with the majority of the literature, all algorithms operate in a continuous search space, with candidate solutions mapped to the binary feature space using the standard threshold θ=0.5.

The implementations of all the considered algorithms were taken from the Niapy framework [21]. To ensure a fair comparison, the population size of all the algorithms was set to 30, along with 3000 maximum function evaluations. Due to the stochastic nature of evolutionary algorithms, each experiment was executed 30 times for each dataset and algorithm. Let us emphasize that the default values of the other algorithm’s parameters, as proposed in the corresponding literature, were employed for the specific algorithms during the experimental work. Because the selected feature selection approach was wrapper-based, the KNN machine learning algorithm was adopted with a value of K=5. This classifier was selected due to its simplicity, computational efficiency, and robustness, which make it particularly suitable for wrapper-based feature selection. KNN has no explicit training phase, allowing for rapid evaluation of candidate feature subsets across many iterations, which is essential in large-scale experimental setups like ours. Furthermore, KNN is used commonly in the literature [22]. By focusing only on the KNN classifier, we ensured that variability in results comes primarily from the threshold adaptation mechanisms and evolutionary algorithms in the study, rather than from differences in classifier behavior. While the threshold adaptation mechanisms may behave differently with classifiers such as Support Vector Machines (SVMs) or Random Forest (RF), the present work deliberately isolates the effect of threshold adaptation. Consequently, all results should be understood as classifier-dependent. In all the described mechanisms, the parameters were set as follows: $\theta_{min}$ was set to 0.1, $\theta_{max}$ was set to 0.9, and target success rate was set to $SR^{★}=0.15$, while the learning rate was set to η=0.05. The choice of fixed θ bounds ensured comparability across datasets, even though these values may have different implications in lower- and higher-dimensional feature spaces.

For evaluating the quality of the feature selection process, the considered metric in Equation (4) was applied using the final feature subset size variable |S|, and the classification accuracy of the selected feature subset parameter was weighted by parameter β. In this study, we tested three values of the weighting factor β, namely, β∈{0.9,0.7,0.5}. The value β=0.9 is a commonly adopted setting in wrapper-based EFS methods [9] and ensures that classification accuracy has a dominant influence in the evaluation of feature subsets, while maintaining a lower weight for subset size. The lower values β=0.7 and β=0.5 were tested, to check whether the lower classification weight (and a higher feature weight) has any significant effect on selecting the final feature subsets. Lower values of β were not considered, as placing a higher weight on the feature subset size can severely degrade algorithm performance by driving the search toward extreme solutions (i.e., selecting almost no features; consequently, the trained classifier fails to capture relevant patterns and achieves low accuracy). Similarly, excessively high β values were avoided to ensure that feature subset size retained at least some influence in the fitness evaluation.

Although we agree that accuracy has limitations, especially in imbalanced or multi-class datasets, the majority of state-of-the-art feature selection studies report accuracy as the primary evaluation metric, which allows us to make a direct comparison with existing works. For consistency and comparability, we therefore adopted accuracy as our main performance measure. Accuracy was computed as the overall classification accuracy across all samples. While alternative metrics such as balanced accuracy, F1-score, or AUC could provide additional insights, incorporating them is beyond the scope of this study and represents an interesting direction for future work.

All the experiments were performed on a desktop computer, with the following configuration:

- Intel(R) Core(TM) i9-10900KF CPU @ 3.70 GHz;
- RAM: 65 GB;
- Operating system: Linux Ubuntu 22.04 Jammy Jellyfish.

To evaluate the impact of a threshold in EFS, the datasets listed in Table 1 were used during the experimental work. The characteristics of each dataset are presented in terms of the number of instances, features, and number of classes. All the datasets contain diverse classification problems, as they contain a different number of instances and features [23]. These datasets are used commonly in the research literature [24].

Each dataset was split randomly into training and testing sets, with 70% of the samples going to the training and 30% to the testing set. When dividing, we paid attention to the equal division of classes between the two sets. One algorithm run consists of selecting the relevant features from the training set and then evaluating the performance of the selected features on the test set. During the training phase, a 5-fold cross-validation scheme was employed on the training set to ensure the robustness and generalizability of the selected feature subsets. Cross-validation is widely recognized as the standard approach to mitigate classifier overfitting in EFS, although it cannot fully eliminate the risk. The test set remained completely unseen throughout the training and feature selection process, and was only used for the final evaluation of the selected features. Let us notice that the datasets were normalized so that no feature with a larger range disproportionately influenced the KNN classifier.

To assess the statistically significant difference between the algorithms in the test, we used the non-parametric Friedman test, which is used for comparing multiple algorithms over multiple datasets by ranks [25,26]. For each dataset, the algorithms were ranked according to their performance. The Friedman statistic was then computed from these ranks and used to test the null hypothesis assuming that all algorithms are equivalent. This means that they have the same expected rank. We performed post hoc analysis only when the Friedman null hypothesis was rejected.

Following Demšar [25], we applied the Nemenyi post hoc test to obtain pairwise comparisons based on average ranks, and to visualize the results with critical difference diagrams, which show which average ranks differ significantly [27]. The Nemenyi procedure is conservative, especially when many algorithms are compared, or when the number of datasets is modest, so its statistical power can be limited, and some pairs may remain indistinguishable [27].

To increase sensitivity, we identified a control method, defined as the algorithm with the lowest average rank, and then applied the Wilcoxon signed-rank test for paired comparisons between each algorithm and the control [26]. This choice follows the recommendation of Benavoli et al., who advocate paired distribution free tests over procedures that rely only on mean ranks, because they offer greater power and a clearer interpretation [28]. In our reporting, the Nemenyi test provides graphical summaries through critical difference diagrams, while the Wilcoxon test provides the primary significance assessment. All the tests were conducted using a significance level α=0.05.

The pairwise observations used in the tests were constructed as follows: For each of the 15 datasets, we considered two summary statistics of the experimental outcomes, namely, the mean and the median. This yielded 2×15=30 pairwise measurements (also classifiers) for each algorithmic comparison and defined the effective sample size for the Wilcoxon analyses reported in the paper.

In the remainder of this section, we illustrate the detailed results of the statistical tests obtained after the conducted experiments.

### 4.1. Determining the Best Baseline Bio-Inspired Algorithm

The purpose of the first experiment was to recognize the best-performing bio-inspired algorithm using the fixed value of the threshold control parameter, which will later be used as a baseline algorithm for comparing against other bio-inspired algorithms using different adaptation mechanisms. The control algorithm will be recognized in terms of the fitness function (Equation (4)), which considers the threshold control parameter to be static throughout the whole evolutionary run of the algorithm that was set to θ=0.5. Given the large number of results, only the aggregated statistical results, considering all datasets, are reported in the corresponding tables.

**Figure 3:**
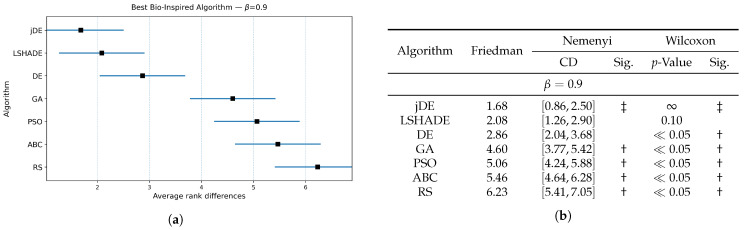
(**a**): Graphical representation of the Friedman critical distances for the best baseline bio-inspired algorithm discovery using β=0.9 and weighting factor β=0.9; (**b**): Friedman and Wilcoxon statistical tests to determine the best baseline bio-inspired algorithm using β=0.9 and weighting factor β=0.9. The ‡ symbol denotes the best-performing method according to the Friedman test, whereas the † indicates a statistically significant difference.

**Figure 4:**
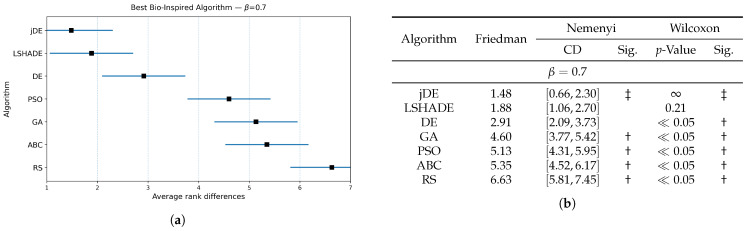
(**a**): Graphical representation of the Friedman critical distances for the best baseline bio-inspired algorithms discovery using β=0.7 and weighting factor β=0.7; (**b**): Friedman and Wilcoxon statistical tests to determine the best baseline bio-inspired algorithm using β=0.7 and weighting factor β=0.7. The ‡ symbol denotes the best-performing method according to the Friedman test, whereas the † indicates a statistically significant difference.

**Figure 5:**
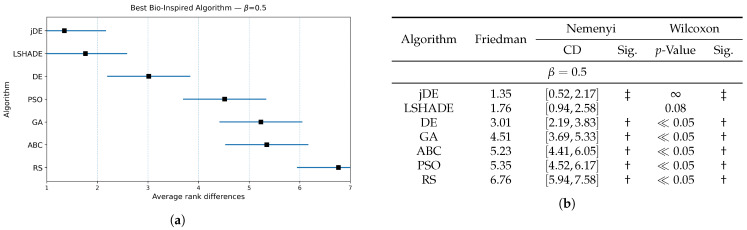
(**a**): Graphical representation of the Friedman critical distances for the best baseline bio-inspired algorithms discovery using β=0.5 and weighting factor β=0.5; (**b**): Friedman and Wilcoxon statistical tests to determine the best baseline bio-inspired algorithm using β=0.5 and weighting factor β=0.5. The ‡ symbol denotes the best-performing method according to the Friedman test, whereas the † indicates a statistically significant difference.

The results of the Friedman and Wilcoxon tests are presented in Figure 3, Figure 4 and Figure 5 for values of weighting factor β=0.9, β=0.7, and β=0.5, respectively. Thus, each figure is divided into two parts, i.e., the table for presenting the results numerically and the diagram for illustrating the same graphically. The table contains the results of the Friedman tests, together with corresponding Nemenyi and Wilcoxon post hoc tests, while the figure presents the calculated Friedman ranks. The results of the Nemenyi post hoc test are represented as critical difference intervals, where the results of two algorithms are statistically significant if their critical difference intervals do not overlap. The Friedman tests’ ranks of particular baseline bio-inspired algorithms were compared with the ranks obtained by the control algorithm, to identify the best bio-inspired algorithm. The results of the Wilcoxon non-parametric test are depicted through corresponding *p*-values, where a significant difference between two algorithms is indicated when p<0.05. In Figure 3b, Figure 4b and Figure 5b, the best baseline algorithm identified by the Nemenyi post hoc test becomes a control algorithm for the Wilcoxon test. The control algorithm serves as a basis for comparison with the other baseline bio-inspired algorithm and is therefore denoted with the symbol ‡ in the table. Moreover, the presence of a significant difference between the control algorithm and the corresponding bio-inspired algorithm is represented by the symbol †.

The Nemenyi post hoc test results are presented graphically through corresponding diagrams in Figure 3a, Figure 4a and Figure 5a. Each diagram displays the average ranks represented by squares, while lines indicate the confidence intervals (critical differences) for the algorithms being compared. Thus, the lower rank values signify better-performing algorithms.

In summary, the jDE baseline bio-inspired algorithm attained the lowest average rank and was taken as the control algorithm. LSHADE was consistently the closest competitor, followed by DE, GA, PSO, ABC, and RS trailing behind. Under the Nemenyi test, the confidence intervals of jDE overlapped with those of LSHADE (and marginally with DE) by using the weighting factors β=0.9 and β=0.7, so these algorithms were not significantly distinguished from the control one, whereas GA, PSO, ABC, and RS were significantly worse. When β=0.5, DE’s interval no longer overlapped with that of jDE; therefore, DE performed significantly worse. LSHADE remained statistically indistinguishable from the control across all three weighting factor values. The Wilcoxon signed-rank test, which has higher power than Nemenyi, corroborated the finding with a stronger separation. For each β, the pairwise Wilcoxon tests indicated significant differences between jDE and the other methods (all p≪0.05), while the differences between jDE and LSHADE were not significant.

Overall, these results suggest a stable two-tier structure, where jDE and LSHADE form the top tier, tied statistically under both post hoc procedures across the tested β values, while DE occupies a borderline position that becomes clearly inferior when the objective places more weight on feature subset size (β=0.5). The algorithms GA, PSO, ABC, and RS constituted the lower tier, being consistently worse than the control under both post hoc analyses. For selecting a baseline optimizer, jDE is therefore a reasonable default, with LSHADE as an equally competitive alternative, depending on the implementation or runtime preferences.

### 4.2. Impact of Different Threshold Parameter Control Mechanisms on the Classification Accuracy

The purpose of this study was to analyze how different threshold parameter control mechanisms influence the classification accuracy. Since the jDE algorithm obtained the best results in the first experiment, it was used as the basic algorithm, whose results should be improved using various threshold parameter controls. Therefore, the jDE algorithm was executed 30 times for each evaluation dataset using five different threshold parameter control mechanisms: LR, CR, SRA, PC, and SA. The obtained classification accuracies and final thresholds are presented in Table 2 for all observed weighting factor values β. In the table, the row marked “# best” indicates the number of datasets on which each mechanism obtained the best results at a specific β value.

Table 2 shows that within jDE, threshold parameter control is most beneficial when accuracy dominates the weighting factor value β=0.9. In line with this, the SA threshold parameter control achieves the most wins (5), while the same algorithm using PC parameter control follows (4), with many best runs converging to higher thresholds (often ≈0.88−0.90). The jDE algorithm incorporating the deterministic parameter control wins only sporadically and the fixed baseline (θ=0.5) tops just a few datasets. As the objective gives more weight to the size of the feature subset (β=0.7), the fixed-baseline algorithm regains ground (6 wins), while the jDE employing the PC parameter control remains a strong, stable second (5). The same algorithm with SA threshold parameter control stays competitive (3), indicating that regulating a target selection rate is often sufficient. At β=0.5, where accuracy and subset size are balanced, results diversify, where the baseline algorithm again leads (6), the algorithm with SA threshold parameter control remains effective on several problems (4), and the same using more reactive mechanisms (SRA and CR) register isolated wins (two each), consistent with scenarios where mid or lower thresholds are preferable. Overall, the jDE applying the SA threshold parameter control offers the highest upside across datasets, while the PC parameter control is the safest default for β∈{0.9,0.7}. We can conclude that deterministic threshold parameter control is inconsistent, while the SRA threshold parameter control is dataset-sensitive.

We can also observe a clear trend that on high-dimensional datasets, the SA and PC threshold parameter controls tend to achieve the best results.

**Figure 6:**
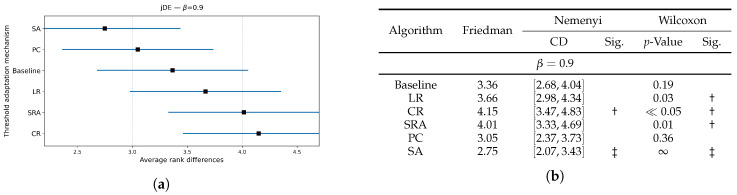
(**a**): Graphical representation of the Friedman critical distances for the classification accuracy using the jDE algorithm with β=0.9 and weighting factor β=0.9; (**b**): Friedman and Wilcoxon statistical tests on classification accuracy using different threshold parameter control mechanisms for the jDE algorithm using β=0.9 and weighting factor β=0.9. The ‡ symbol denotes the best-performing method according to the Friedman test, whereas the † indicates a statistically significant difference.

**Figure 7:**
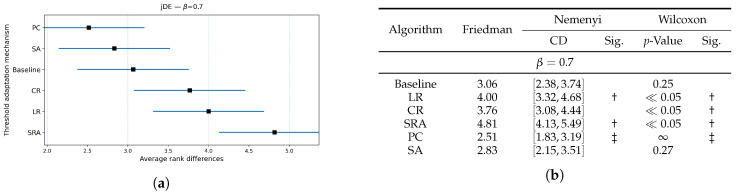
(**a**): Graphical representation of the Friedman critical distances for the classification accuracy using the jDE algorithm with β=0.7 and weighting factor β=0.7; (**b**): Friedman and Wilcoxon statistical tests on classification accuracy using different threshold parameter control mechanisms for the jDE algorithm using β=0.7 and weighting factor β=0.7. The ‡ symbol denotes the best-performing method according to the Friedman test, whereas the † indicates a statistically significant difference.

**Figure 8:**
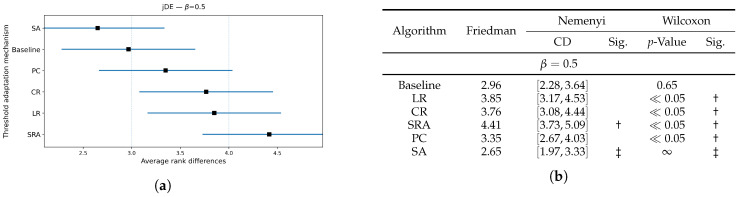
(**a**): Graphical representation of the Friedman critical distances for the classification accuracy using the jDE algorithm with β=0.5 and weighting factor β=0.5; (**b**): Friedman and Wilcoxon statistical tests on classification accuracy using different threshold parameter control mechanisms for the jDE algorithm using β=0.5 and weighting factor β=0.5. The ‡ symbol denotes the best-performing method according to the Friedman test, whereas the † indicates a statistically significant difference.

Figure 6, Figure 7 and Figure 8 extend the jDE analysis by comparing threshold control parameter mechanisms at three weighting factor values β. The Friedman test determined the SA threshold parameter control as the best method at β=0.9 and β=0.5, and PC threshold parameter control at β=0.7. By using the weighting factor value β=0.9, the Friedman test did not declare other threshold parameter control mechanisms significantly different from SA, while Wilcoxon detected that the LR, CR, and SRA threshold parameter control mechanisms were significantly worse (all p≤0.03), whereas the Baseline and PC threshold parameter control mechanisms were not (p=0.19 and 0.36). At β=0.7, the PC threshold parameter control mechanism clearly leads, since the Friedman and Wilcoxon tests both separated it from the LR, CR, and SRA threshold parameter control mechanisms (all significant), and the SA threshold parameter control mechanism remained statistically comparable (Wilcoxon p=0.27). At β=0.5, the SA threshold parameter control mechanism again attained the best rank, where the post hoc Nemenyi test marked the SRA threshold parameter control mechanism as significantly worse. Additionally, the results of the Wilcoxon pairwise non-parametric statistical tests reported the PC, SRA, LR, and CR threshold parameter control mechanisms as worse (p≪0.05), while the Baseline remained indistinguishable (p=0.65). Overall, the SA per-individual threshold parameter control mechanism was the most reliable choice, when accuracy dominates, or is balanced with feature subset size in the fitness function, while the PC threshold parameter control mechanism was preferred at the intermediate setting (β=0.7).

### 4.3. Impact of the Feature Threshold Parameter Control Mechanisms on the Feature Subset Size

The purpose of this study was to analyze the effect of different feature threshold parameter control mechanisms on the size of the final feature subset. The obtained feature subset sizes, along with final thresholds, are presented in Table 3 for all β values.

Table 3 reports the selected feature subset sizes for jDE using different threshold parameter control mechanisms and three values of weighting factor values β={0.9,0.7,0.5}. Overall, the jDE using the adaptive parameter control mechanisms shrank the subset noticeably compared to the Baseline jDE algorithm, with the best count row # best indicating that the PC threshold parameter control dominated when accuracy was emphasized (β=0.9, 9 wins), while the SA threshold parameter control became progressively stronger as the feature subset size importance increased (β=0.7: 6 wins; β=0.5: 8 wins). At weighting factor value β=0.9, the jDE incorporating the PC threshold parameter control achieved large reductions on high-dimensional datasets (e.g., BrainTumor1: 2764→1104; LungCancer: 6084→2338), while the same algorithm with the SA threshold parameter control was close behind. The deterministic threshold parameter controls incorporated into the jDE algorithm reduce the feature subset size but less aggressively, while the SRA threshold parameter control was occasionally unstable, even inflating the subset (e.g., UrbanLandCover: 48.9→81.0). Considering β=0.7, the jDE using the SA threshold parameter control was best on most large feature datasets (for example, BrainTumor1: 877; LungCancer: 1609), while the PC threshold parameter control remained a strong second. The determinstic schedules (LR and CR) were best on a few smaller datasets (e.g., Musk1, HillValley). With the weighting factor value β=0.5, the jDE employing the SA threshold parameter control was the clear winner by searching for the minimum subset size. It reached the smallest subset sizes on the majority of datasets (e.g., ProstateTumor1: 611 vs. baseline 2745; BrainTumor1: 681 vs. 2734), while PC still provided substantial reductions.

Across all weighting factor values β, it was notable that datasets with fewer features (e.g., German, Segmentation, Ionosphere) naturally hit small absolute feature set sizes, sometimes matching the baseline floor, whereas on high-dimensional datasets the gains from adaptive threshold parameter control are both larger in magnitude and typically lower in variance (see, for instance, Isolet5 and Madelon). By using the jDE algorithm, the PC threshold parameter control was better suited when accuracy predominates, while the SA threshold parameter control produces better results as the fitness function places more weight on the number of selected features.

**Figure 9:**
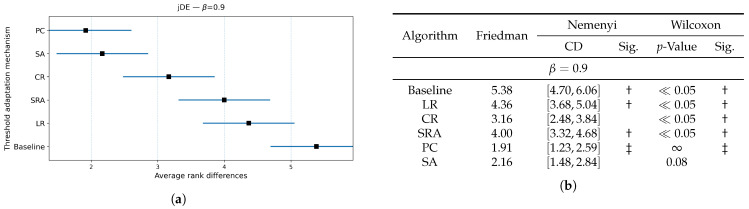
(**a**): Graphical representation of the Friedman critical distances for the feature subset size using the jDE algorithm with weighting factor value β=0.9 and weighting factor β=0.9; (**b**): Friedman and Wilcoxon statistical tests on feature subset size using different threshold parameter control mechanisms for the jDE algorithm using weighting factor value β=0.9 and weighting factor β=0.9. The ‡ symbol denotes the best-performing method according to the Friedman test, whereas the † indicates a statistically significant difference.

**Figure 10:**
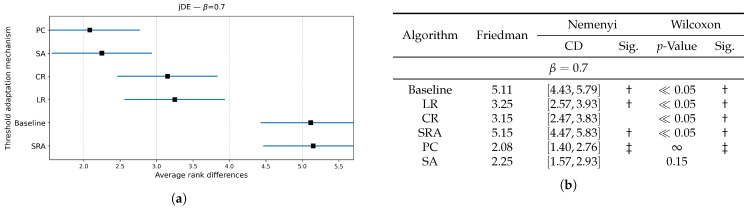
(**a**): Graphical representation of the Friedman critical distances for the feature subset size using the jDE algorithm with weighting factor value β=0.7 and weighting factor β=0.7; (**b**): Friedman and Wilcoxon statistical tests on feature subset size using different threshold parameter control mechanisms for the jDE algorithm using weighting factor value β=0.7 and weighting factor β=0.7. The ‡ symbol denotes the best-performing method according to the Friedman test, whereas the † indicates a statistically significant difference.

**Figure 11:**
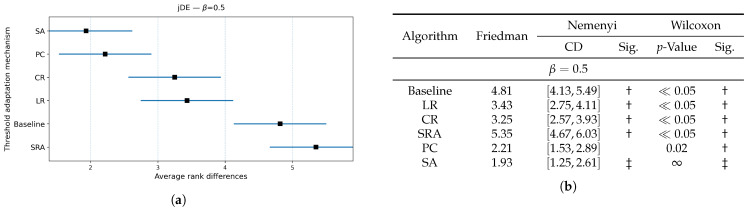
(**a**): Graphical representation of the Friedman critical distances for the feature subset size using the jDE algorithm with weighting factor value β=0.5, Weighting factor β=0.5; (**b**): Friedman and Wilcoxon statistical tests on feature subset size using different threshold parameter control mechanisms for the jDE algorithm using weighting factor value β=0.5, Weighting factor β=0.5. The ‡ symbol denotes the best-performing method according to the Friedman test, whereas the † indicates a statistically significant difference.

Figure 9, Figure 10 and Figure 11 compared threshold parameter control mechanisms built in the jDE algorithm with respect to the size of the selected feature subset for all observed weighting factor values β={0.9,0.7,0.5}. When accuracy dominated (when β={0.9,0.7}), the PC threshold parameter control attained the best average rank, while the SA threshold parameter control was statistically indistinguishable from it according to the Wilcoxon non-parametric statistical test, whereas the Baseline jDE and the jDE using the LR, CR, and SRA threshold parameter controls yielded significantly larger subsets (all p≪0.05). As the objective placed more weight on limiting the number of selected features (when β=0.5), the jDE incorporating the SA threshold parameter control became the best and delivered the smallest subsets consistently. The same algorithm using the PC threshold parameter control remained the closest competitor but produced significantly larger subset sizes according to the Wilcoxon non-parametric statistical test. Across all the weighting factor values β, the jDE using deterministic threshold parameter controls (i.e., LR and CR) reduced the feature subset sizes relative to the Baseline jDE algorithm, but remained significantly worse than the jDE with the SA threshold parameter control. The SRA threshold parameter control was distinguished as the most unreliable, often producing the largest subsets. In short, a global target on the selection rate by the PC threshold parameter control is sufficient when accuracy is important, while by the SA per-individual threshold parameter control, it becomes decisively superior as the fitness function rewards smaller feature subsets increasingly.

### 4.4. Convergence Analysis

**Figure 12:**
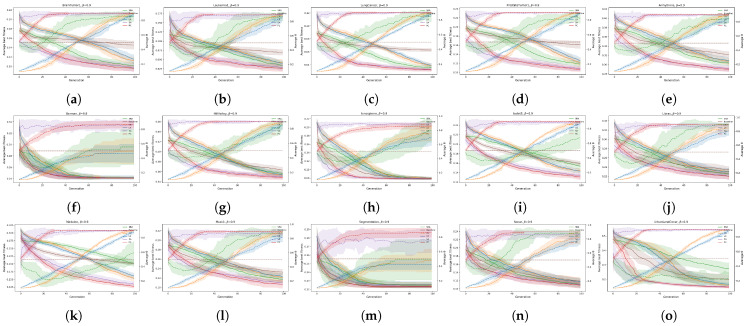
Convergence analysis of fitness and θ for jDE and all datasets for β = 0.9: (**a**): BrainTumor1, (**b**): Leukemia1, (**c**): LungCancer, (**d**): ProstateTumor1, (**e**): Arrhythmia, (**f**): German, (**g**): HillValley, (**h**): Ionosphere, (**i**): Isolet5, (**j**): Libras, (**k**): Madelon, (**l**): Musk1, (**m**): Segmentation, (**n**): Sonar, and (**o**): UrbanLandCover.

**Figure 13:**
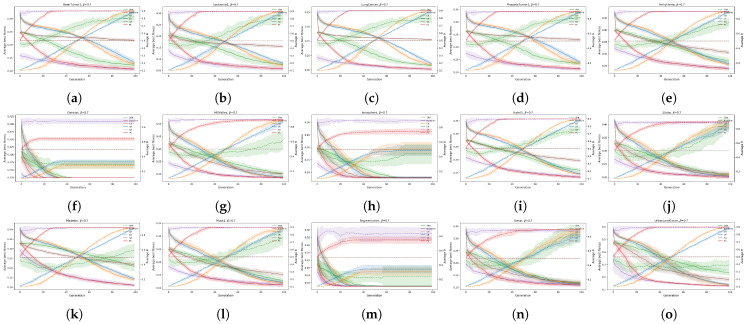
Convergence analysis of fitness and θ for jDE and all datasets for β = 0.7: (**a**): BrainTumor1, (**b**): Leukemia1, (**c**): LungCancer, (**d**): ProstateTumor1, (**e**): Arrhythmia, (**f**): German, (**g**): HillValley, (**h**): Ionosphere, (**i**): Isolet5, (**j**): Libras, (**k**): Madelon, (**l**): Musk1, (**m**): Segmentation, (**n**): Sonar, and (**o**): UrbanLandCover.

**Figure 14:**
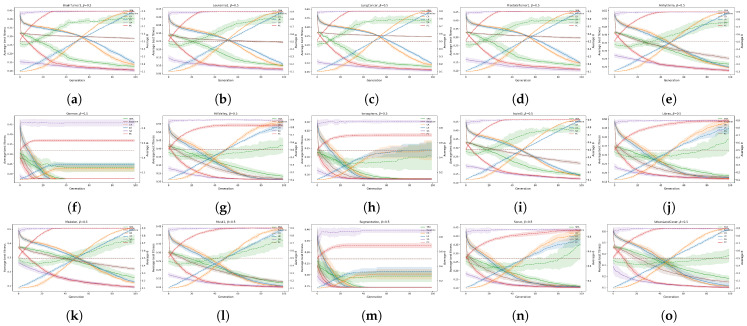
Convergence analysis of fitness and θ for jDE and all datasets for β = 0.5: (**a**): BrainTumor1, (**b**): Leukemia1, (**c**): LungCancer, (**d**): ProstateTumor1, (**e**): Arrhythmia, (**f**): German, (**g**): HillValley, (**h**): Ionosphere, (**i**): Isolet5, (**j**): Libras, (**k**): Madelon, (**l**): Musk1, (**m**): Segmentation, (**n**): Sonar, and (**o**): UrbanLandCover.

The mentioned experiment was reserved for comparing the convergence rates of different bio-inspired algorithms for wrapper-based feature selection considering different feature selection threshold parameter controls. The convergence rates are presented in terms of fitness convergence (see Figure 12, Figure 13 and Figure 14) and by using the Generation to Convergence Metric (GTC), which is defined as the generation number at which the best fitness value was first obtained during the run of the evolutionary algorithm. This metric captures how quickly an algorithm is able to reach its “optimal” solution, providing additional insights into its convergence speed. The results of the GTC metric are reported in Table 4.

Let us notice that mechanisms that previously delivered strong accuracy and smaller feature subsets also tended to converge in fewer generations, although the balance depends on the weighting factor β. At the weighting factor value β=0.9, the jDE armed with the SA threshold parameter control most often reached convergence earliest, for example, on BrainTumor1, Leukemia1, LungCancer, HillValley, Ionosphere, Sonar, and UrbanLandCover. This aligns with its accuracy gains and near-minimal subset sizes at this setting. The jDE using the PC threshold parameter control was close to the former and was best on some datasets, e.g., Musk1 and Madelon. When the fitness function placed more weight on limiting the number of selected features, that is β=0.7, the same algorithm with the PC threshold parameter control frequently achieved the lowest GTC on high-dimensional datasets in the first block of datasets, while those using the CR threshold parameter control converged fastest on several medium-size datasets, e.g., Ionosphere, Isolet5, Libras, Musk1, Segmentation, and Sonar. This mirrors the earlier results, where the jDE using the SA threshold parameter control minimized subset size, but using the PC or the CR threshold parameter control often reached convergence earlier, which indicates a balance between speed and compactness. The selected algorithm incorporated with PC threshold parameter control was best on Leukemia1, ProstateTumor1, Libras, Madelon, Musk1, Segmentation, and UrbanLandCover, while the same using the SA threshold parameter control was best on BrainTumor1 and LungCancer. The CR threshold parameter control was distinguished as the fastest on a few datasets, such as Arrhythmia, HillValley, and Ionosphere.

Across all the observed weighted factor values β, the jDE using the SRA threshold parameter control minimized the GTC rarely. The Baseline jDE algorithm occasionally converged most quickly on very low-dimensional datasets, such as German and Segmentation at β=0.9, which was consistent with the smaller search space. For early convergence with competitive accuracy, the jDE armed with the PC threshold parameter control is a safe default, especially when β=0.7 or β=0.5. The SA threshold parameter control offers similar or better convergence speed at β=0.9 and remains attractive when the goal is fast convergence, strong accuracy, and small feature subsets. Deterministic threshold parameter controls can accelerate convergence on some datasets but they should be weighed against their subpar performance.

The fitness convergence rates are reported in Figure 12, Figure 13 and Figure 14 for all values of the weighting factors β={0.9,0.7,0.5}, where the solid lines present the average fitness, and the dotted lines represent the average θ for each dataset.

### 4.5. Comparison of the Best Threshold Parameter Control Mechanism with the State-of-the-Art Algorithms

To assess the effectiveness and external validity of the proposed threshold parameter control mechanisms, we compared our best-performing bio-inspired algorithm (i.e., the jDE using the SA threshold parameter control) with three representative state-of-the-art algorithms from the literature. We selected studies that reported average classification accuracy under wrapper-based settings and extracted the published mean classification accuracy and standard deviations. The comparison was restricted to datasets that overlap with ours, to ensure a like-for-like evaluation. One of the selected papers [9] departed from the conventional fixed threshold θ=0.5 and used θ=0.6, with the same fitness function formulation and the weighting factor value β=0.9. The other two papers [29,30] implemented method-specific procedures within PSO or DE, and used a fixed threshold value θ=0.5. For each study, we ran a paired Wilcoxon non-parametric signed-rank statistical test, with the null hypothesis that the results of the jDE with the SA threshold parameter control and the method taken from literature perform equally. The resulting *p*-values in Table 5 indicate statistically significant improvements in favor of the jDE using the SA threshold parameter control, including against the method increasing the fixed threshold to a value of θ=0.6. These findings suggest that modifying the threshold during the evolutionary run provides a measurable advantage over the fixed-threshold designs and over method-specific heuristics. We note that the original studies may differ in train and test data partitioning. Despite this heterogeneity, the direction and magnitude of the changes are consistent across the overlapping datasets, which supports the threshold parameter control as a default component in wrapper-based EFS.

### 4.6. Discussion

The results support feature threshold parameter control consistently as a key design choice in wrapper-based EFS. In the algorithm-level comparison, the jDE algorithm emerged as the stronger baseline, the LSHADE was statistically indistinguishable from it in several settings, and the DE, GA, PSO, ABC, and RS algorithms ranked lower according to both the Nemenyi and Wilcoxon non-parametric statistical tests. Built in jDE, the obtained accuracies show that the SA threshold parameter control attained the largest number of per-dataset wins at a weighting factor value of β=0.9, while the same one using the PC threshold parameter control was a close second. As the weight factor on the feature subset size was modified to β=0.7 and β=0.5, the jDE using the PC threshold parameter control remained competitive, while the same algorithm armed with the SA threshold parameter control continued to produce the best results. Deterministic threshold parameter control in jDE can be useful but rarely dominated, while the SRA threshold parameter control was the most sensitive to short-term improvement.

The subset size analysis aligned with these trends. When accuracy dominated at the weighting factor value β={0.9,0.7}, the jDE using the PC threshold parameter control produced the smallest sets on most high-dimensional datasets, while the SA threshold parameter control was not statistically different from it. When the fitness function placed more emphasis on limiting the number of selected features, the jDE using the SA threshold parameter control became the best choice and won most frequently at the weighting factor β=0.5. The deterministic threshold parameter controls reduced the feature subsets relative to the baseline jDE algorithm but remained significantly worse than the best mechanism, while the SRA threshold parameter control often yielded the largest feature subsets. These patterns confirm that a global target on the selection rate is effective when accuracy is the priority, whereas the SA per-individual threshold parameter control becomes advantageous once feature subset size is more important.

The results also show that the obtained accuracies tend to vary only slightly across independent runs, whereas the size of the selected feature subsets exhibits greater variability. This difference can be attributed to the stochastic initialization of the algorithms, which encourages exploration of diverse regions of the search space. Importantly, despite fluctuations in the subset size, classification accuracy remained stable, suggesting that different subsets found can yield comparably good performance.

The convergence analysis results are consistent with the accuracy and feature subset size findings. At the weighting factor value β=0.9, the jDE using the SA threshold parameter control most often converged in fewer generations and did so while maintaining better accuracy and smaller feature subsets. At the weighting factor values β=0.7 and β=0.5, the jDE with built-in PC threshold parameter control frequently converged fastest on large datasets, while the same one using the CR threshold parameter control can be the fastest on some medium-size datasets. The fixed-baseline jDE algorithm can converge quickly on very low-dimensional datasets, which was expected given the small search space. The jDE using the SRA threshold parameter control rarely minimized the number of generations and can be unstable in terms of convergence.

A comparison with the state-of-the-art algorithm results also confirms these conclusions. Using the overlapping datasets and the reported means and standard deviations from three representative studies, the Wilcoxon non-parametric statistical test shows that the jDE with the SA threshold parameter control mechanism achieved significantly higher accuracy than all three references at the weighting factor value β=0.9. This includes a method that had already improved over the conventional setting by fixing θ=0.6. The direction of the differences was consistent across the shared datasets, despite minor differences in the evaluation protocols.

While the results demonstrate the effectiveness of threshold parameter control in wrapper-based EFS, several limitations of the present study should be noted. First, although cross-validation was employed to mitigate classifier overfitting, wrapper-based approaches remain vulnerable in extremely high-dimensional datasets with limited samples, where the risk of overfitting cannot be fully eliminated. Second, certain datasets exhibit substantial class imbalance, which may bias the KNN classifier toward majority classes and affect reported accuracy. Cross-validation reduces but does not remove these biases completely. Finally, it is important to note that the obtained results may not generalize directly to more complex classifiers.

## 5. Conclusions

This study examined different threshold adaptation mechanisms in wrapper-based EFS for classification. We evaluated five referenced bio-inspired algorithms and a random search method on widely used benchmark datasets. The analysis covered three aspects of performance, namely, classification accuracy, the size of the selected feature subset, and the number of generations to convergence. By holding the objective and the evaluation protocol fixed, we isolated the effect of threshold control from other algorithmic factors.

The results show that threshold adaptation in feature selection should be considered a default design choice. Within jDE, which emerged as a strong baseline with LSHADE as a close alternative, SA achieved the highest classification accuracy, most often when classification accuracy had a higher weight in the fitness function. As the fitness function placed more weight on limiting the number of selected features, SA also produced the smallest subsets most frequently. The algorithm also converged faster when using the SA mechanism and a higher weight on classification in the fitness function, whereas PC often converged earlier on larger datasets when the feature subset size gained more importance.

A comparison with representative state-of-the-art methods on overlapping datasets supports these conclusions further. By using statistical tests on the reported results, SA obtained statistically significant better results. This held even when the literature already improved the fixed threshold by moving from the default value to a larger constant. The combination of internal benchmarks and external comparisons therefore indicates that adapting the threshold during the evolutionary run provides measurable benefits over fixed-threshold designs.

For future research, we want to test more recent bio-inspired algorithms and use even larger datasets. It would also be interesting to investigate hybrid adaptation mechanisms that combine a global target with per-individual threshold mechanisms, and to study per-feature threshold adaptation. Another priority is a multi-objective formulation of the problem that treats accuracy and feature subset size as separate objectives. In addition, extending the study beyond the KNN classifier would strengthen the generalizability of the findings and reveal whether threshold adaptation interacts differently with classifiers of varying complexity. Finally, theoretical analysis of the stability of threshold dynamics and their interaction with population diversity would deepen the understanding of when and why adaptation is useful.

## Figures and Tables

**Table 1:** Experimental datasets used in the study.

Dataset	Features	Instances	Classes
Arrhythmia	279	452	13
German	20	1000	2
HillValley	100	1212	2
Ionosphere	34	351	2
Isolet5	617	1559	26
Libras	90	360	15
Madelon	500	2600	2
Musk1	166	476	2
Segmentation	19	210	7
Sonar	60	208	2
UrbanLandCover	147	675	9
BrainTumor1	5920	90	5
ProstateTumor1	5966	102	2
LungCancer	12,600	203	5
Leukemia1	5327	72	3

**Table 2:** Results of the average classification accuracies for all datasets using all threshold parameter control mechanisms and the basic jDE algorithm for all the observed weighting factor values β. The best-performing threshold parameter control mechanism for each dataset is highlighted in bold.

Dataset	Baseline	LR	CR	SRA	PC	SA
β=0.9						
BrainTumor1	0.838 ± 0.03 (0.50 ± 0.00)	0.844 ± 0.02 (0.88 ± 0.03)	0.831 ± 0.04 (0.89 ± 0.01)	0.841 ± 0.03 (0.85 ± 0.07)	**0.852 ± 0.03 (0.90 ± 0.00)**	0.832 ± 0.03 (0.90 ± 0.01)
Leukemia1	0.876 ± 0.03 (0.50 ± 0.00)	0.868 ± 0.06 (0.88 ± 0.04)	0.880 ± 0.05 (0.88 ± 0.03)	0.892 ± 0.05 (0.87 ± 0.06)	0.883 ± 0.06 (0.90 ± 0.00)	**0.898 ± 0.05 (0.90 ± 0.00)**
LungCancer	0.907 ± 0.02 (0.50 ± 0.00)	0.911 ± 0.02 (0.88 ± 0.03)	0.910 ± 0.02 (0.89 ± 0.02)	0.911 ± 0.02 (0.84 ± 0.07)	0.910 ± 0.03 (0.90 ± 0.00)	**0.913 ± 0.02 (0.90 ± 0.01)**
ProstateTumor1	0.889 ± 0.03 (0.50 ± 0.00)	0.894 ± 0.04 (0.89 ± 0.01)	0.897 ± 0.04 (0.90 ± 0.01)	0.890 ± 0.05 (0.83 ± 0.07)	**0.910 ± 0.03 (0.90 ± 0.00)**	0.910 ± 0.04 (0.90 ± 0.01)
Arrhythmia	**0.627 ± 0.03 (0.50 ± 0.00)**	0.617 ± 0.03 (0.84 ± 0.05)	0.607 ± 0.04 (0.89 ± 0.02)	0.616 ± 0.03 (0.85 ± 0.07)	0.601 ± 0.03 (0.90 ± 0.00)	0.611 ± 0.04 (0.89 ± 0.01)
German	0.734 ± 0.01 (0.50 ± 0.00)	0.733 ± 0.02 (0.47 ± 0.13)	0.732 ± 0.01 (0.50 ± 0.18)	0.731 ± 0.02 (0.56 ± 0.25)	**0.735 ± 0.01 (0.87 ± 0.05)**	0.734 ± 0.01 (0.86 ± 0.06)
HillValley	0.552 ± 0.01 (0.50 ± 0.00)	0.548 ± 0.01 (0.85 ± 0.05)	0.551 ± 0.01 (0.88 ± 0.03)	0.550 ± 0.01 (0.86 ± 0.07)	0.555 ± 0.01 (0.90 ± 0.00)	**0.561 ± 0.01 (0.89 ± 0.01)**
Ionosphere	0.917 ± 0.03 (0.50 ± 0.00)	**0.922 ± 0.04 (0.69 ± 0.11)**	0.915 ± 0.04 (0.76 ± 0.11)	0.919 ± 0.04 (0.79 ± 0.17)	0.909 ± 0.04 (0.86 ± 0.04)	0.919 ± 0.03 (0.89 ± 0.02)
Isolet5	0.848 ± 0.01 (0.50 ± 0.00)	0.842 ± 0.01 (0.86 ± 0.04)	0.841 ± 0.01 (0.89 ± 0.02)	0.838 ± 0.01 (0.67 ± 0.15)	0.850 ± 0.02 (0.90 ± 0.00)	**0.852 ± 0.01 (0.88 ± 0.02)**
Libras	**0.741 ± 0.02 (0.50 ± 0.00)**	0.730 ± 0.02 (0.80 ± 0.07)	0.737 ± 0.02 (0.85 ± 0.06)	0.732 ± 0.03 (0.90 ± 0.02)	0.736 ± 0.03 (0.90 ± 0.00)	0.733 ± 0.02 (0.89 ± 0.02)
Madelon	0.786 ± 0.02 (0.50 ± 0.00)	0.813 ± 0.02 (0.88 ± 0.03)	0.823 ± 0.02 (0.90 ± 0.01)	0.778 ± 0.02 (0.59 ± 0.16)	**0.846 ± 0.01 (0.90 ± 0.00)**	0.839 ± 0.01 (0.90 ± 0.00)
Musk1	0.866 ± 0.03 (0.50 ± 0.00)	0.864 ± 0.04 (0.82 ± 0.07)	0.857 ± 0.03 (0.88 ± 0.02)	0.873 ± 0.03 (0.88 ± 0.05)	0.869 ± 0.04 (0.90 ± 0.00)	**0.879 ± 0.03 (0.87 ± 0.06)**
Segmentation	0.812 ± 0.01 (0.50 ± 0.00)	**0.818 ± 0.02 (0.42 ± 0.07)**	0.812 ± 0.01 (0.48 ± 0.16)	0.813 ± 0.02 (0.49 ± 0.28)	0.807 ± 0.02 (0.86 ± 0.06)	0.813 ± 0.01 (0.75 ± 0.16)
Sonar	**0.817 ± 0.04 (0.50 ± 0.00)**	0.802 ± 0.04 (0.80 ± 0.08)	0.809 ± 0.05 (0.82 ± 0.09)	0.798 ± 0.04 (0.88 ± 0.05)	0.802 ± 0.05 (0.90 ± 0.00)	0.800 ± 0.04 (0.86 ± 0.05)
UrbanLandCover	0.830 ± 0.04 (0.50 ± 0.00)	**0.842 ± 0.02 (0.87 ± 0.02)**	0.835 ± 0.02 (0.90 ± 0.00)	0.762 ± 0.08 (0.23 ± 0.08)	0.840 ± 0.02 (0.90 ± 0.00)	0.837 ± 0.02 (0.89 ± 0.01)
β=0.7						
BrainTumor1	**0.836 ± 0.02 (0.50 ± 0.00)**	0.826 ± 0.04 (0.89 ± 0.01)	0.821 ± 0.04 (0.90 ± 0.00)	0.822 ± 0.03 (0.82 ± 0.07)	0.836 ± 0.03 (0.90 ± 0.00)	0.825 ± 0.04 (0.90 ± 0.00)
Leukemia1	0.874 ± 0.03 (0.50 ± 0.00)	0.858 ± 0.06 (0.89 ± 0.02)	0.892 ± 0.05 (0.89 ± 0.01)	0.879 ± 0.06 (0.80 ± 0.06)	**0.903 ± 0.05 (0.90 ± 0.00)**	0.885 ± 0.04 (0.90 ± 0.00)
LungCancer	0.905 ± 0.02 (0.50 ± 0.00)	0.916 ± 0.02 (0.90 ± 0.01)	0.914 ± 0.02 (0.90 ± 0.00)	0.904 ± 0.02 (0.79 ± 0.05)	0.914 ± 0.02 (0.90 ± 0.00)	**0.920 ± 0.03 (0.90 ± 0.00)**
ProstateTumor1	0.882 ± 0.04 (0.50 ± 0.00)	0.908 ± 0.03 (0.89 ± 0.01)	0.890 ± 0.05 (0.90 ± 0.00)	0.891 ± 0.03 (0.82 ± 0.07)	**0.918 ± 0.04 (0.90 ± 0.00)**	0.908 ± 0.04 (0.90 ± 0.00)
Arrhythmia	**0.619 ± 0.03 (0.50 ± 0.00)**	0.582 ± 0.06 (0.89 ± 0.02)	0.595 ± 0.05 (0.90 ± 0.01)	0.610 ± 0.04 (0.75 ± 0.06)	0.585 ± 0.05 (0.90 ± 0.00)	0.589 ± 0.04 (0.90 ± 0.00)
German	**0.713 ± 0.01 (0.50 ± 0.00)**	0.707 ± 0.02 (0.33 ± 0.03)	0.709 ± 0.02 (0.28 ± 0.04)	0.695 ± 0.04 (0.29 ± 0.05)	0.701 ± 0.02 (0.64 ± 0.02)	0.711 ± 0.01 (0.88 ± 0.04)
HillValley	0.552 ± 0.01 (0.50 ± 0.00)	0.557 ± 0.01 (0.87 ± 0.03)	0.558 ± 0.01 (0.89 ± 0.01)	0.551 ± 0.01 (0.62 ± 0.12)	**0.560 ± 0.01 (0.89 ± 0.01)**	0.556 ± 0.01 (0.90 ± 0.01)
Ionosphere	0.954 ± 0.01 (0.50 ± 0.00)	0.934 ± 0.03 (0.48 ± 0.07)	0.934 ± 0.03 (0.51 ± 0.09)	0.920 ± 0.03 (0.44 ± 0.14)	0.954 ± 0.01 (0.74 ± 0.04)	**0.957 ± 0.00 (0.89 ± 0.02)**
Isolet5	**0.846 ± 0.01 (0.50 ± 0.00)**	0.837 ± 0.02 (0.89 ± 0.01)	0.833 ± 0.02 (0.90 ± 0.00)	0.825 ± 0.02 (0.76 ± 0.05)	0.845 ± 0.02 (0.90 ± 0.00)	0.843 ± 0.02 (0.90 ± 0.00)
Libras	0.735 ± 0.02 (0.50 ± 0.00)	0.729 ± 0.03 (0.82 ± 0.06)	0.725 ± 0.03 (0.86 ± 0.05)	0.724 ± 0.04 (0.87 ± 0.08)	**0.740 ± 0.02 (0.90 ± 0.01)**	0.735 ± 0.03 (0.89 ± 0.02)
Madelon	0.796 ± 0.01 (0.50 ± 0.00)	0.823 ± 0.01 (0.90 ± 0.01)	0.829 ± 0.02 (0.90 ± 0.00)	0.779 ± 0.02 (0.60 ± 0.09)	**0.854 ± 0.01 (0.90 ± 0.00)**	0.852 ± 0.01 (0.90 ± 0.00)
Musk1	0.863 ± 0.04 (0.50 ± 0.00)	0.856 ± 0.03 (0.88 ± 0.03)	0.853 ± 0.03 (0.88 ± 0.04)	0.864 ± 0.03 (0.76 ± 0.10)	0.874 ± 0.04 (0.90 ± 0.00)	**0.876 ± 0.03 (0.90 ± 0.01)**
Segmentation	0.809 ± 0.00 (0.50 ± 0.00)	**0.815 ± 0.01 (0.34 ± 0.05)**	0.815 ± 0.01 (0.31 ± 0.05)	0.812 ± 0.01 (0.24 ± 0.14)	0.815 ± 0.01 (0.75 ± 0.05)	0.809 ± 0.00 (0.83 ± 0.09)
Sonar	**0.810 ± 0.06 (0.50 ± 0.00)**	0.785 ± 0.05 (0.80 ± 0.07)	0.797 ± 0.06 (0.86 ± 0.05)	0.791 ± 0.06 (0.83 ± 0.11)	0.795 ± 0.06 (0.90 ± 0.00)	0.791 ± 0.06 (0.89 ± 0.02)
UrbanLandCover	**0.837 ± 0.03 (0.50 ± 0.00)**	0.819 ± 0.04 (0.89 ± 0.02)	0.834 ± 0.02 (0.90 ± 0.00)	0.777 ± 0.10 (0.39 ± 0.07)	0.831 ± 0.02 (0.90 ± 0.00)	0.834 ± 0.02 (0.90 ± 0.00)
β=0.5						
BrainTumor1	**0.828 ± 0.04 (0.50 ± 0.00)**	0.826 ± 0.04 (0.90 ± 0.00)	0.810 ± 0.04 (0.90 ± 0.01)	0.820 ± 0.03 (0.80 ± 0.06)	0.820 ± 0.03 (0.90 ± 0.00)	0.828 ± 0.05 (0.90 ± 0.00)
Leukemia1	0.883 ± 0.02 (0.50 ± 0.00)	0.892 ± 0.05 (0.90 ± 0.01)	0.873 ± 0.04 (0.90 ± 0.00)	**0.894 ± 0.05 (0.81 ± 0.07)**	0.877 ± 0.06 (0.90 ± 0.00)	0.888 ± 0.04 (0.90 ± 0.00)
LungCancer	0.903 ± 0.02 (0.50 ± 0.00)	0.909 ± 0.02 (0.90 ± 0.00)	**0.920 ± 0.02 (0.90 ± 0.01)**	0.904 ± 0.02 (0.77 ± 0.03)	0.913 ± 0.02 (0.90 ± 0.00)	0.907 ± 0.03 (0.90 ± 0.00)
ProstateTumor1	0.887 ± 0.04 (0.50 ± 0.00)	0.891 ± 0.04 (0.90 ± 0.01)	0.900 ± 0.04 (0.90 ± 0.00)	0.898 ± 0.04 (0.80 ± 0.06)	0.910 ± 0.03 (0.90 ± 0.00)	**0.923 ± 0.03 (0.90 ± 0.00)**
Arrhythmia	**0.604 ± 0.03 (0.50 ± 0.00)**	0.593 ± 0.04 (0.89 ± 0.01)	0.593 ± 0.04 (0.90 ± 0.00)	0.601 ± 0.04 (0.76 ± 0.06)	0.587 ± 0.04 (0.90 ± 0.00)	0.585 ± 0.04 (0.90 ± 0.00)
German	**0.713 ± 0.01 (0.50 ± 0.00)**	0.689 ± 0.04 (0.30 ± 0.03)	0.706 ± 0.03 (0.26 ± 0.04)	0.690 ± 0.03 (0.27 ± 0.06)	0.701 ± 0.04 (0.63 ± 0.02)	0.704 ± 0.01 (0.87 ± 0.04)
HillValley	0.556 ± 0.01 (0.50 ± 0.00)	0.556 ± 0.01 (0.83 ± 0.06)	**0.557 ± 0.01 (0.87 ± 0.04)**	0.544 ± 0.01 (0.56 ± 0.10)	0.556 ± 0.01 (0.82 ± 0.02)	0.554 ± 0.01 (0.90 ± 0.01)
Ionosphere	0.945 ± 0.03 (0.50 ± 0.00)	0.926 ± 0.04 (0.50 ± 0.10)	0.924 ± 0.04 (0.50 ± 0.13)	0.922 ± 0.03 (0.42 ± 0.19)	0.927 ± 0.04 (0.70 ± 0.02)	**0.947 ± 0.02 (0.89 ± 0.02)**
Isolet5	**0.832 ± 0.02 (0.50 ± 0.00)**	0.808 ± 0.02 (0.89 ± 0.01)	0.819 ± 0.02 (0.90 ± 0.00)	0.806 ± 0.02 (0.78 ± 0.04)	0.827 ± 0.02 (0.90 ± 0.00)	0.828 ± 0.02 (0.90 ± 0.00)
Libras	**0.732 ± 0.03 (0.50 ± 0.00)**	0.715 ± 0.05 (0.79 ± 0.07)	0.706 ± 0.04 (0.87 ± 0.05)	0.700 ± 0.04 (0.66 ± 0.18)	0.707 ± 0.03 (0.89 ± 0.01)	0.719 ± 0.03 (0.89 ± 0.01)
Madelon	0.804 ± 0.02 (0.50 ± 0.00)	0.825 ± 0.02 (0.89 ± 0.01)	0.833 ± 0.01 (0.90 ± 0.00)	0.787 ± 0.02 (0.75 ± 0.06)	0.857 ± 0.01 (0.90 ± 0.00)	**0.860 ± 0.01 (0.90 ± 0.00)**
Musk1	0.850 ± 0.04 (0.50 ± 0.00)	0.839 ± 0.04 (0.87 ± 0.03)	0.837 ± 0.03 (0.89 ± 0.01)	0.842 ± 0.03 (0.74 ± 0.09)	0.850 ± 0.04 (0.90 ± 0.00)	**0.857 ± 0.03 (0.90 ± 0.00)**
Segmentation	0.784 ± 0.01 (0.50 ± 0.00)	0.772 ± 0.04 (0.33 ± 0.06)	0.784 ± 0.02 (0.29 ± 0.07)	**0.786 ± 0.01 (0.26 ± 0.07)**	0.782 ± 0.02 (0.68 ± 0.03)	0.786 ± 0.00 (0.88 ± 0.03)
Sonar	0.783 ± 0.06 (0.50 ± 0.00)	0.789 ± 0.06 (0.76 ± 0.08)	0.792 ± 0.06 (0.83 ± 0.07)	0.787 ± 0.08 (0.70 ± 0.20)	**0.802 ± 0.07 (0.88 ± 0.02)**	0.796 ± 0.07 (0.89 ± 0.02)
UrbanLandCover	**0.830 ± 0.02 (0.50 ± 0.00)**	0.813 ± 0.04 (0.89 ± 0.01)	0.819 ± 0.02 (0.90 ± 0.00)	0.785 ± 0.09 (0.54 ± 0.09)	0.823 ± 0.02 (0.90 ± 0.00)	0.826 ± 0.02 (0.90 ± 0.00)
# best	3/6/6	3/1/0	0/0/2	0/0/2	4/5/1	5/3/4

**Table 3:** Result of the average feature subset sizes for all datasets obtained by the Baseline jDE algorithm and the jDE using different threshold parameter control mechanisms for all observed weighting factor values β. The best-performing threshold parameter control mechanism for each dataset is highlighted in bold.

Dataset	Baseline	LR	CR	SRA	PC	SA
β=0.9						
BrainTumor1	2764.43 ± 55.92	1443.17 ± 171.71	1324.83 ± 126.47	1404.70 ± 323.90	**1103.50 ± 127.25**	1108.93 ± 139.76
Leukemia1	2471.73 ± 67.89	1284.63 ± 167.55	1225.20 ± 188.58	1259.77 ± 251.19	**1016.70 ± 109.03**	1068.30 ± 108.17
LungCancer	6084.33 ± 73.23	2905.17 ± 300.60	2832.83 ± 309.65	3152.43 ± 681.21	**2337.73 ± 185.42**	2453.80 ± 229.31
ProstateTumor1	2784.73 ± 60.62	1257.97 ± 130.21	1264.23 ± 94.00	1525.17 ± 368.47	1057.43 ± 135.87	**1047.87 ± 115.46**
Arrhythmia	114.93 ± 5.28	63.10 ± 14.14	55.07 ± 8.61	62.83 ± 13.74	47.90 ± 8.14	**47.70 ± 6.13**
German	**5.00 ± 0.00**	5.53 ± 0.73	5.53 ± 0.90	5.73 ± 1.23	5.23 ± 0.82	5.00 ± 0.00
HillValley	26.50 ± 3.84	16.50 ± 2.93	**14.30 ± 3.01**	14.80 ± 4.88	15.27 ± 2.56	14.87 ± 2.78
Ionosphere	3.63 ± 0.89	3.57 ± 1.01	**3.23 ± 0.77**	3.53 ± 1.22	3.60 ± 1.00	3.60 ± 0.81
Isolet5	266.73 ± 14.28	132.23 ± 17.80	126.87 ± 17.42	208.37 ± 58.55	**123.53 ± 10.90**	126.73 ± 10.02
Libras	30.37 ± 4.31	20.27 ± 4.50	19.63 ± 2.93	18.20 ± 3.72	**17.70 ± 2.88**	17.70 ± 3.25
Madelon	207.57 ± 9.89	88.73 ± 15.39	78.77 ± 13.10	191.73 ± 49.86	**56.57 ± 10.47**	63.33 ± 9.75
Musk1	68.83 ± 6.47	41.37 ± 7.50	36.00 ± 5.13	35.87 ± 6.27	**34.70 ± 6.20**	37.07 ± 6.71
Segmentation	**5.00 ± 0.00**	5.50 ± 0.73	5.47 ± 1.01	5.80 ± 1.32	5.20 ± 0.41	5.00 ± 0.00
sonar	20.47 ± 2.66	13.93 ± 3.41	13.30 ± 3.64	13.03 ± 3.30	**11.20 ± 1.88**	12.70 ± 2.96
UrbanLandCover	48.90 ± 5.35	23.33 ± 5.14	21.90 ± 4.24	80.97 ± 9.04	**18.80 ± 3.01**	18.80 ± 3.70
β=0.7						
BrainTumor1	2781.43 ± 55.97	1190.13 ± 195.41	1146.97 ± 112.59	1449.40 ± 332.07	937.40 ± 126.68	**877.10 ± 147.00**
Leukemia1	2476.07 ± 63.20	1116.00 ± 118.75	1042.90 ± 119.06	1421.53 ± 259.56	912.10 ± 115.75	**899.83 ± 121.39**
LungCancer	6036.00 ± 63.68	2313.83 ± 215.98	2074.77 ± 311.04	3092.90 ± 733.62	1626.23 ± 293.06	**1608.93 ± 279.72**
ProstateTumor1	2775.90 ± 47.32	1118.93 ± 113.32	1098.67 ± 115.78	1463.50 ± 320.89	874.07 ± 154.77	**870.37 ± 123.33**
Arrhythmia	78.93 ± 5.73	30.03 ± 6.05	30.40 ± 5.73	55.43 ± 10.99	**23.90 ± 4.98**	24.23 ± 4.52
german	**1.00 ± 0.00**	1.47 ± 0.68	1.43 ± 0.73	2.10 ± 1.18	1.23 ± 0.43	1.00 ± 0.00
HillValley	11.00 ± 2.88	5.27 ± 1.64	**4.77 ± 1.25**	11.03 ± 5.21	5.13 ± 1.78	5.30 ± 1.06
Ionosphere	2.17 ± 0.38	2.70 ± 1.02	2.73 ± 0.94	3.37 ± 1.13	2.23 ± 0.43	**2.03 ± 0.18**
Isolet5	211.23 ± 8.70	89.33 ± 8.60	88.87 ± 10.30	150.23 ± 25.68	86.03 ± 9.76	**85.43 ± 9.77**
Libras	15.43 ± 2.21	10.90 ± 2.28	10.90 ± 2.12	11.03 ± 2.41	**10.57 ± 1.98**	11.60 ± 2.47
Madelon	161.40 ± 9.86	60.07 ± 9.62	61.23 ± 7.83	166.93 ± 29.93	**39.53 ± 5.46**	39.70 ± 8.76
Musk1	43.33 ± 4.00	**20.80 ± 4.21**	21.73 ± 3.77	31.00 ± 7.74	21.77 ± 3.70	23.17 ± 2.97
Segmentation	**4.00 ± 0.00**	4.37 ± 0.67	4.60 ± 0.81	4.73 ± 1.11	4.43 ± 0.63	4.00 ± 0.00
Sonar	9.00 ± 1.74	**6.43 ± 1.33**	6.53 ± 1.33	7.37 ± 1.96	6.57 ± 1.38	6.67 ± 1.18
UrbanLandCover	27.07 ± 4.23	11.27 ± 2.73	11.27 ± 2.49	50.73 ± 7.96	**9.07 ± 2.27**	9.90 ± 1.63
β=0.5						
BrainTumor1	2733.80 ± 48.22	1024.07 ± 109.36	935.63 ± 129.98	1265.80 ± 293.25	685.50 ± 121.96	**680.73 ± 114.62**
Leukemia1	2454.00 ± 44.53	942.60 ± 110.39	858.63 ± 94.50	1163.17 ± 334.25	717.20 ± 113.81	**645.60 ± 115.98**
LungCancer	5981.47 ± 56.50	1983.07 ± 219.26	1815.50 ± 271.64	2842.97 ± 461.63	1208.43 ± 171.96	**1120.00 ± 232.41**
ProstateTumor1	2745.30 ± 45.51	927.20 ± 136.08	987.60 ± 92.71	1271.60 ± 354.65	676.30 ± 86.29	**610.77 ± 121.28**
Arrhythmia	49.83 ± 4.84	17.57 ± 4.26	18.10 ± 3.12	45.10 ± 9.94	**12.80 ± 3.41**	12.87 ± 2.58
German	**1.00 ± 0.00**	1.50 ± 0.78	1.60 ± 0.77	2.47 ± 1.53	1.13 ± 0.35	1.00 ± 0.00
HillValley	3.53 ± 1.04	3.23 ± 0.73	3.20 ± 0.55	8.70 ± 3.20	3.03 ± 0.18	**3.00 ± 0.26**
Ionosphere	**1.97 ± 0.18**	2.50 ± 0.94	2.83 ± 1.29	3.03 ± 1.52	2.03 ± 0.18	2.00 ± 0.00
Isolet5	168.37 ± 9.60	70.00 ± 7.30	68.17 ± 7.60	126.37 ± 22.38	59.90 ± 6.55	**58.83 ± 8.97**
Libras	8.63 ± 1.59	7.30 ± 1.47	**6.77 ± 1.04**	10.03 ± 3.18	6.83 ± 0.91	7.33 ± 1.06
Madelon	131.83 ± 8.79	47.60 ± 6.25	46.17 ± 7.68	109.63 ± 21.71	29.23 ± 5.42	**28.57 ± 4.26**
Musk1	21.93 ± 2.88	**10.83 ± 2.10**	11.97 ± 1.88	22.63 ± 5.72	12.10 ± 2.12	13.33 ± 2.59
Segmentation	**2.00 ± 0.00**	2.50 ± 0.73	2.70 ± 0.99	2.67 ± 0.80	2.27 ± 0.58	2.00 ± 0.00
Sonar	5.20 ± 1.00	4.93 ± 1.17	4.73 ± 1.39	4.90 ± 2.04	4.67 ± 1.18	**4.60 ± 1.30**
UrbanLandCover	16.77 ± 2.82	6.53 ± 1.50	6.23 ± 1.63	31.80 ± 6.16	**5.00 ± 0.91**	5.87 ± 1.20
# best	2/2/3	0/2/1	2/1/1	0/0/0	9/4/2	2/6/8

**Table 4:** Results of the GTC metric for all datasets and weighting factor values β, by using different feature threshold parameter control mechanisms built in the jDE algorithm. The best-performing threshold parameter control mechanism for each dataset is highlighted in bold.

Dataset	Baseline	LR	SRA	SA	CR	PC
β=0.9						
BrainTumor1	92.6 ± 7.1	95.4 ± 3.8	86.4 ± 11.3	**85.4 ± 14.5**	95.1 ± 4.2	86.7 ± 12.8
Leukemia1	89.6 ± 8.8	95.3 ± 5.4	91.7 ± 6.5	**87.5 ± 11.3**	92.7 ± 7.2	87.8 ± 10.0
LungCancer	90.2 ± 10.8	95.3 ± 3.2	89.6 ± 8.4	**87.4 ± 11.2**	94.2 ± 5.0	89.7 ± 9.6
ProstateTumor1	92.2 ± 7.0	97.6 ± 1.4	**87.6 ± 9.7**	89.0 ± 9.4	95.7 ± 3.2	88.0 ± 10.6
Arrhythmia	91.5 ± 7.7	90.7 ± 6.8	89.2 ± 8.4	**87.8 ± 9.6**	92.9 ± 5.1	88.0 ± 10.2
German	**32.9 ± 10.5**	44.5 ± 15.8	53.3 ± 15.1	39.9 ± 19.3	48.8 ± 15.7	40.1 ± 18.4
HillValley	91.1 ± 9.0	91.6 ± 6.5	91.9 ± 6.9	**82.8 ± 12.6**	91.2 ± 7.4	87.9 ± 9.4
Ionosphere	67.9 ± 16.4	69.6 ± 13.8	79.0 ± 13.2	**49.2 ± 19.6**	72.8 ± 12.4	52.6 ± 19.7
Isolet5	93.5 ± 4.8	93.8 ± 4.5	93.1 ± 4.3	**91.9 ± 8.7**	92.6 ± 5.8	94.2 ± 5.5
Libras	**83.5 ± 16.2**	86.2 ± 8.3	89.8 ± 7.4	85.7 ± 12.1	84.7 ± 10.1	84.1 ± 13.7
Madelon	93.4 ± 5.5	95.6 ± 3.3	95.2 ± 3.5	94.0 ± 5.7	95.4 ± 4.0	**93.4 ± 4.8**
Musk1	86.1 ± 11.5	87.7 ± 8.4	90.3 ± 8.8	87.0 ± 9.8	91.2 ± 6.5	**84.4 ± 10.1**
Segmentation	**31.9 ± 12.3**	38.6 ± 8.2	45.7 ± 15.4	38.2 ± 12.9	47.6 ± 15.8	32.5 ± 15.4
Sonar	86.5 ± 14.1	85.4 ± 9.9	88.8 ± 8.4	**81.5 ± 15.3**	82.5 ± 13.0	85.2 ± 13.0
UrbanLandCover	94.8 ± 5.3	95.0 ± 3.1	95.6 ± 4.6	**86.9 ± 13.6**	95.8 ± 2.8	89.1 ± 8.1
β=0.7						
BrainTumor1	92.0 ± 6.9	97.7 ± 2.0	96.7 ± 2.1	91.2 ± 7.6	90.3 ± 7.2	**80.9 ± 14.5**
Leukemia1	91.9 ± 10.9	96.9 ± 2.3	93.7 ± 4.2	90.9 ± 8.2	87.9 ± 9.8	**83.0 ± 15.8**
LungCancer	93.3 ± 7.4	98.0 ± 1.2	96.7 ± 2.2	90.8 ± 5.2	90.4 ± 8.4	**83.9 ± 12.5**
ProstateTumor1	93.5 ± 4.8	97.3 ± 2.0	97.0 ± 2.0	91.4 ± 7.5	89.3 ± 9.3	**84.3 ± 10.5**
Arrhythmia	94.7 ± 4.1	97.2 ± 2.2	96.4 ± 3.0	94.6 ± 4.7	90.5 ± 8.8	**89.5 ± 9.7**
German	17.7 ± 4.2	27.1 ± 3.8	29.8 ± 3.9	34.5 ± 6.3	10.9 ± 2.6	**5.0 ± 3.2**
HillValley	90.6 ± 5.3	93.7 ± 3.8	92.8 ± 5.3	95.5 ± 3.9	84.0 ± 15.0	**82.6 ± 14.5**
Ionosphere	43.6 ± 10.8	46.6 ± 9.0	49.6 ± 7.1	65.3 ± 6.3	**33.0 ± 14.0**	33.1 ± 13.7
Isolet5	96.8 ± 2.5	96.9 ± 2.1	95.7 ± 2.9	93.3 ± 4.2	**90.9 ± 7.0**	93.4 ± 6.2
Libras	89.1 ± 6.9	87.8 ± 7.7	86.5 ± 10.2	90.8 ± 6.1	**77.0 ± 14.8**	81.6 ± 16.4
Madelon	97.3 ± 2.5	97.9 ± 1.4	97.2 ± 1.6	95.7 ± 2.8	95.2 ± 3.6	**92.8 ± 11.3**
Musk1	91.7 ± 9.0	96.0 ± 3.8	91.1 ± 7.6	92.3 ± 6.5	**84.5 ± 10.9**	86.1 ± 11.8
Segmentation	22.1 ± 7.3	29.0 ± 6.7	32.6 ± 4.9	41.5 ± 11.2	**19.9 ± 6.0**	24.2 ± 8.8
Sonar	88.7 ± 10.2	86.0 ± 9.2	86.3 ± 9.1	88.0 ± 7.3	**79.5 ± 17.8**	84.9 ± 13.2
UrbanLandCover	95.1 ± 5.0	96.8 ± 2.6	96.2 ± 2.3	95.4 ± 3.3	93.3 ± 4.7	**92.6 ± 6.3**
β=0.5						
BrainTumor1	91.6 ± 8.9	98.5 ± 0.9	95.9 ± 3.4	**88.4 ± 8.9**	89.7 ± 13.9	88.4 ± 8.0
Leukemia1	95.4 ± 8.1	98.0 ± 1.2	96.2 ± 2.6	91.0 ± 6.4	89.3 ± 10.4	**85.0 ± 12.6**
LungCancer	92.4 ± 7.1	98.4 ± 0.7	96.4 ± 3.0	**88.3 ± 9.5**	89.8 ± 11.6	88.5 ± 13.6
ProstateTumor1	93.4 ± 6.9	98.1 ± 1.1	97.2 ± 1.8	90.8 ± 7.2	89.9 ± 6.5	**84.3 ± 17.5**
Arrhythmia	96.0 ± 2.6	97.9 ± 1.9	96.2 ± 2.3	93.2 ± 5.9	**91.5 ± 7.9**	91.8 ± 6.8
German	15.8 ± 4.3	24.1 ± 3.4	27.8 ± 4.5	31.8 ± 7.1	11.1 ± 2.8	**5.1 ± 3.6**
HillValley	91.8 ± 7.3	88.2 ± 8.0	87.8 ± 8.1	94.9 ± 4.4	**74.7 ± 14.2**	75.7 ± 13.2
Ionosphere	44.9 ± 15.9	48.8 ± 12.2	49.3 ± 13.0	64.7 ± 12.4	**30.9 ± 10.7**	33.7 ± 16.5
Isolet5	96.4 ± 2.6	97.2 ± 1.9	96.8 ± 1.9	**93.3 ± 6.0**	94.1 ± 4.3	94.5 ± 5.5
Libras	87.7 ± 9.5	84.1 ± 8.6	88.7 ± 8.8	92.9 ± 5.1	79.9 ± 14.6	**77.9 ± 18.9**
Madelon	96.0 ± 2.8	97.6 ± 1.8	96.8 ± 2.4	96.1 ± 3.1	95.7 ± 3.5	**94.5 ± 5.5**
Musk1	94.3 ± 4.6	94.7 ± 3.5	94.6 ± 4.7	94.4 ± 5.5	90.0 ± 8.4	**85.7 ± 13.0**
Segmentation	18.9 ± 6.0	27.3 ± 7.4	31.0 ± 6.1	35.5 ± 7.7	14.7 ± 4.4	**14.3 ± 5.5**
Sonar	87.6 ± 8.8	80.9 ± 10.1	82.0 ± 11.4	90.7 ± 5.9	**75.3 ± 15.2**	77.3 ± 16.3
UrbanLandCover	96.7 ± 2.4	97.2 ± 2.0	96.2 ± 2.9	95.5 ± 3.9	93.8 ± 4.6	**91.0 ± 7.1**

**Table 5:** Comparison of the best overall proposed jDE using the SA threshold parameter control mechanism with the state-of-the-art algorithms according to average classification accuracy.

Method	Bio-Inspired Algorithm	Included Datasets	Method Specifics	Wilcox Test (*p*-Value)
[9]	PSO	Sonar, Libras, HillValley, UrbanLandCover, Musk1, Arrhythmia, Madelon, Isolet5	Binary encoded features, θ=0.6, β=0.9	0.015
[29]	DE	Sonar, Musk1, Madelon, Isolet5	θ=0.5, β=0.9	0.039
[30]	DE	German, Ionsophere, Sonar, Libras, HillValley, UrbanLandCover, Musk1	θ=0.5, β=0.9	0.021

## Data Availability

The data used in this study is available on request from the corresponding author.

## References

[1] Wang L., Wang Y., Chang Q. (2016). Feature selection methods for big data bioinformatics: A survey from the search perspective. Methods.

[2] Nguyen B.H., Xue B., Zhang M. (2020). A survey on swarm intelligence approaches to feature selection in data mining. Swarm Evol. Comput..

[3] Kirkpatrick S., Gelatt C.D., Vecchi M.P. (1983). Optimization by Simulated Annealing. Science.

[4] Hoque N., Bhattacharyya D.K., Kalita J.K. (2014). MIFS-ND: A mutual information-based feature selection method. Expert Syst. Appl..

[5] Eiben A.E., Smith J.E. (2015). Introduction to Evolutionary Computing.

[6] Blum C., Merkle D. (2008). Swarm Intelligence: Introduction and Applications.

[7] Deng S., Li Y., Wang J., Cao R., Li M. (2023). A feature-thresholds guided genetic algorithm based on a multi-objective feature scoring method for high-dimensional feature selection. Appl. Soft Comput..

[8] Kononenko I. Estimating attributes: Analysis and extensions of RELIEF. Proceedings of the European Conference on Machine Learning.

[9] Li A.D., Xue B., Zhang M. (2021). Improved binary particle swarm optimization for feature selection with new initialization and search space reduction strategies. Appl. Soft Comput..

[10] Fister D., Fister I., Jagrič T., Fister I., Brest J. (2018). A novel self-adaptive differential evolution for feature selection using threshold mechanism. Proceedings of the 2018 IEEE Symposium Series on Computational Intelligence (SSCI).

[11] Fister I., Brest J., Mlakar U., Fister I. Towards the universal framework of stochastic nature-inspired population-based algorithms. Proceedings of the 2016 IEEE Symposium Series on Computational Intelligence (SSCI).

[12] Hancer E., Xue B., Zhang M. (2018). Differential evolution for filter feature selection based on information theory and feature ranking. Knowl.-Based Syst..

[13] Osei-Kwakye J., Han F., Amponsah A.A., Ling Q.H., Abeo T.A. (2023). A diversity enhanced hybrid particle swarm optimization and crow search algorithm for feature selection. Appl. Intell..

[14] Brest J., Greiner S., Boskovic B., Mernik M., Zumer V. (2006). Self-adapting control parameters in differential evolution: A comparative study on numerical benchmark problems. IEEE Trans. Evol. Comput..

[15] Tanabe R., Fukunaga A.S. (2014). Improving the search performance of SHADE using linear population size reduction. Proceedings of the 2014 IEEE Congress on Evolutionary Computation (CEC).

[16] Karaboğa D. (2005). An Idea Based on Honey Bee Swarm for Numerical Optimization.

[17] Agrawal P., Abutarboush H.F., Ganesh T., Mohamed A.W. (2021). Metaheuristic algorithms on feature selection: A survey of one decade of research (2009–2019). IEEE Access.

[18] Dokeroglu T., Deniz A., Kiziloz H.E. (2022). A comprehensive survey on recent metaheuristics for feature selection. Neurocomputing.

[19] Abu Khurma R., Aljarah I., Sharieh A., Abd Elaziz M., Damaševičius R., Krilavičius T. (2022). A review of the modification strategies of the nature inspired algorithms for feature selection problem. Mathematics.

[20] Anderson R.L. (1953). Recent Advances in Finding Best Operating Conditions. J. Am. Stat. Assoc..

[21] Vrbančič G., Brezočnik L., Mlakar U., Fister D., Fister I. (2018). NiaPy: Python microframework for building nature-inspired algorithms. J. Open Source Softw..

[22] Rostami M., Berahmand K., Nasiri E., Forouzandeh S. (2021). Review of swarm intelligence-based feature selection methods. Eng. Appl. Artif. Intell..

[23] Dua D., Graff C. (2017). UCI Machine Learning Repository. https://archive.ics.uci.edu/.

[24] Song X., Zhang Y., Zhang W., He C., Hu Y., Wang J., Gong D. (2024). Evolutionary computation for feature selection in classification: A comprehensive survey of solutions, applications and challenges. Swarm Evol. Comput..

[25] Demšar J. (2006). Statistical comparisons of classifiers over multiple data sets. J. Mach. Learn. Res..

[26] Rey D., Neuhäuser M. (2011). Wilcoxon-Signed-Rank Test. International Encyclopedia of Statistical Science.

[27] Nemenyi P. (1963). Distribution-Free Multiple Comparisons. Ph.D. Thesis.

[28] Benavoli A., Corani G., Mangili F. (2016). Should we really use post-hoc tests based on mean-ranks?. J. Mach. Learn. Res..

[29] Hancer E. (2019). Differential evolution for feature selection: A fuzzy wrapper–filter approach. Soft Comput..

[30] Hancer E., Xue B., Zhang M. (2022). Fuzzy filter cost-sensitive feature selection with differential evolution. Knowl.-Based Syst..

